# Advancing Dementia Risk Prediction and Prevention in Africa Through Precision Health Approaches

**DOI:** 10.1002/alz70860_105510

**Published:** 2025-12-23

**Authors:** Chinedu T Udeh‐Momoh

**Affiliations:** ^1^ Wake Forest University School of Medicine, Winston‐Salem, NC, USA; Brain and Mind Institute, Aga Khan University, Nairobi Province, Nairobi, Kenya

## Abstract

Africa's vast genetic, cultural, and environmental diversity presents unique challenges and opportunities for dementia risk reduction. With over 60% of projected dementia cases by 2050 expected in low‐ and middle‐income countries (LMICs), including those in the continent, contextually relevant, evidence‐based strategies are urgently needed, yet Africa remains critically underrepresented in prevention research. Despite the continent's rich diversity, most dementia risk prediction models are derived from non‐African populations, limiting their generalizability generalizability and applicability to African contexts. Africa‐FINGERS, the first precision prevention clinical trial for dementia risk reduction in Africa, seeks to address this gap by integrating validated dementia risk scores with biomarker‐informed early detection and culturally tailored interventions.

Building on the **WW‐FINGERS** framework that features the landmark FINGER model, Africa‐FINGERS incorporates culturally informed multidomain intervention strategies providing cognitive enrichment, cardiometabolic risk management, healthy nutrition, and social engagement. The program employs community‐based system dynamics approaches to ensure stakeholder engagement and long‐term sustainability. For its inception, Vanguard epidemiological cohorts across Kenya (AD‐Detect Kenya, FemBER‐Africa, Brain Resilience Kenya, LOSHAK) and Nigeria (VALIANT, PERODEM, ADiBio, Northern Nigeria Dementia Project) provide deeply‐phenotyped dementia risk markers. These cohorts integrate neuroimaging (**MRI, FDG‐PET**), fluid biomarkers (CSF, blood, saliva), and locally adapted neuropsychological assessments to optimize trial enrollment and targeted interventions.

Preliminary analyses demonstrate high concordance between traditional dementia risk scores and biomarker profiles in African populations, validating their predictive accuracy following necessary contextual modifications. Hypertension, elevated fasting blood glucose, and social isolation emerge as key modifiable risk factors, highlighting critical intervention targets for dementia prevention. Furthermore, deep immunophenotyping and multiplex AD biomarker assays provide novel insights into disease pathways, informing precision‐targeted strategies.

The Africa‐FINGERS trial is designed to align with global dementia prevention initiatives, including US‐POINTER, LatAm‐FINGERS, and WW‐FINGERS, enabling comparative cross‐cohort analyses and bridging African and diaspora populations to uncover shared and unique dementia risk factors. This program pioneers a scalable, biomarker‐driven model for dementia prevention in LMICs, advancing brain health equity in underrepresented populations. Africa‐FINGERS underscores the urgency of inclusive precision prevention approaches, positioning Africa as a critical player in the global dementia research landscape.

